# Clinical Perspectives on the Safety Profile of Poly‐L‐Lactic Acid (Juläine): Results From a National Survey

**DOI:** 10.1111/jocd.70439

**Published:** 2025-09-08

**Authors:** Maribel Serrano‐Coronado, Paula Catena Rallo, Francisca Rubio Toral, Cristina Chantada Tirado, Adriana Ribé Subirà, Victoria Páez Ruiz

**Affiliations:** ^1^ Clínica Tufet Barcelona Spain; ^2^ Clínica Paula Catena Madrid Spain; ^3^ Clínica Toral Barcelona Spain; ^4^ Clínica Dr Chantada Málaga Spain; ^5^ Clínica Ribé Barcelona Spain; ^6^ Clínica Dra Páez Málaga Spain

To the Editor,

Poly‐L‐lactic acid (PLLA), a biocompatible and biodegradable polymer with over 30 years of safety data [1], has been shown to be effective in volume augmentation, wrinkle reduction, and treating HIV‐associated lipoatrophy [1, 2, 3, 4, 5, 6]. Targeted applications in supraperiosteal, subcutaneous, and subdermal layers provide optimal results [2, 3, 4, 5, 6]. Safety studies report mostly mild‐to‐moderate adverse events (AEs), such as bruising and tenderness, with rare severe AEs [2, 3, 4, 5, 6]. A systematic review of 1801 patients found 92.3% had no complications. Mild complications occurred in 5.1%, moderate in 2.4%, and severe in only 0.2% of cases [4].

This article presents the findings of a national survey conducted among aesthetic practitioners to assess their clinical perceptions about the safety profile of a next‐generation injectable PLLA‐LaSynPro (Juläine, Nordberg Medical AB, Sweden). This innovative formulation was designed to stimulate collagen production, offering advanced solutions for aesthetic treatments.

A multidisciplinary team of six experts in aesthetic medicine designed a survey to evaluate clinical perceptions about the safety profile of this novel PLLA‐LaSynPro filler (A detailed description of the study methodology is provided as Data S1).

The questionnaire included 18 questions, addressing professional background and perceptions of the safety profile, specifically the risk of AEs incidence (Table S1 shows the English version of the survey).

The survey targeted aesthetic practitioners with experience using the new PLLA‐LaSynPro filler. It was distributed to 48 practitioners, with a pre‐study requirement that at least 36 responses be collected to achieve a significance level of 0.05 and a margin of error of 10%.

A total of 36 professionals responded to the survey. Of these, 8 (22.2%) had used the product for < 3 months, 17 (47.2%) for 3 to 6 months, and 11 (30.6%) had > 6 months of experience with the new PLLA‐LaSynPro filler (Table 1).

Regarding incidence, short duration edema and short‐duration pain were perceived as “very frequent” or “frequent” by > 25% of respondents. Conversely, hyperpigmentation, long duration edema, infection, ulceration, itching, nodules (both early and late onset), cramps at the injection site, ischemic events, and visual impairment/blindness were classified as “rare” or “never observed” by 90% or more of the respondents (Figure 1, Table 1).

**FIGURE 1:**
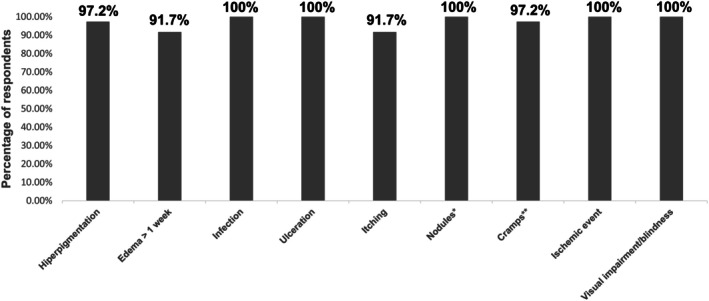
Adverse events (AEs) perceived as “rare” or “never observed” by ≥ 90% of the respondents. The percentages represent the proportion of respondents who classified the adverse event as “rare” or “never observed.” *Both early and late onset (≤ 3 months) and late (> 3 months). **Cramps in the injection area.

**TABLE 1:** Clinical perception of adverse event incidence rates: survey response overview.

Question	Answers, *n* (%); [95% CI]
Q1. Time of experience with PLLA, months	
< 3	8 (22.2)
3–6	17 (47.2)
> 6	11 (30.6)
Q2. Hyperpigmentation	
Very frequent	0 (0.0); [0–10.3]
Frequent	1 (2.8); [0–15.5]
Uncommon	0 (0.0); [0–10.3]
Rare	2 (5.6); [0.7–20.1]
Never observed	33 (91.7); [63.1–100]
Q3. Loss of pigmentation	
Very frequent	1 (2.8); [0–15.5]
Frequent	1 (2.8); [0–15.5]
Uncommon	3 (8.3); [1.8–24.4]
Rare	2 (5.6); [0.7–20.1]
Never observed	29 (80.6); [54.0–100]
Q4. Short‐duration edema (< 1 week)	
Very frequent	7 (19.4); [7.8–40.1]
Frequent	10 (27.8); [13.3–51.1]
Uncommon	9 (25.0); [11.4–47.5]
Rare	8 (22.0); [9.6–43.8]
Never observed	2 (5.6); [0.7–20.1]
Q5. Long duration edema (> 1 week)	
Very frequent	0 (0.0); [0–10.3]
Frequent	1 (2.8); [0–15.5]
Uncommon	2 (5.6); [0.7–20.1]
Rare	5 (13.9); [4.5–32.4]
Never observed	28 (77.8); [51.7–100]
Q6. Short duration pain (< 1 week)	
Very frequent	4 (11.1); [3.0–28.5]
Frequent	5 (13.9); [4.5–32.4]
Uncommon	5 (13.9); [4.5–32.4]
Rare	12 (33.3); [17.2–58.2]
Never observed	10 (27.8); [13.3–51.1]
Q7. Infection	
Very frequent	0 (0.0); [0–10.3]
Frequent	0 (0.0); [0–10.3]
Uncommon	0 (0.0); [0–10.3]
Rare	2 (5.6); [0.7–20.1]
Never observed	34 (94.4); [65.4–100]
Q8. Mild bleeding	
Very frequent	2 (5.6); [0.7–20.1]
Frequent	1 (2.8); [0–15.5]
Uncommon	3 (8.3); [1.8–24.4]
Rare	8 (22.2); [9.6–43.8]
Never observed	22 (61.1); [38.3–92.5]
Q9. Redness/Erythema	
Very frequent	1 (2.8); [0–15.5]
Frequent	6 (16.7); [6.1–36.3]
Uncommon	10 (27.8); [13.3–51.1]
Rare	10 (27.8); [13.3–51.1]
Never observed	9 (25.0); [11.4–47.5]
Q10. Bruising/Hematomas	
Very frequent	0 (0.0); [0–10.3]
Frequent	2 (5.6); [0.7–20.1]
Uncommon	10 (27.8); [13.3–51.1]
Rare	16 (44.4); [25.4–72.2]
Never observed	8 (22.2); [9.6–43.8]
Q11. Ulceration	
Very frequent	0 (0.0); [0–10.3]
Frequent	0 (0.0); [0–10.3]
Uncommon	0 (0.0); [0–10.3]
Rare	1 (2.8); [0–15.5]
Never observed	35 (97.2); [67.7–100]
Q12. Itching	
Very frequent	0 (0.0); [0–10.3]
Frequent	3 (8.3); [1.8–24.4]
Uncommon	0 (0.0); [0–10.3]
Rare	4 (11.1); [3.0–28.5]
Never observed	29 (80.6); [54.0–100]
Q13. Early onset nodules (≤ 3 months)^a^	
Very frequent	0 (0.0); [0–10.5]
Frequent	0 (0.0); [0–10.5]
Uncommon	0 (0.0); [0–10.5]
Rare	1 (2.9); [0.1–15.9]
Never observed	34 (97.1); [67.3–100]
Q14. Late onset nodules (> 3 months)	
Very frequent	0 (0.0); [0–10.3]
Frequent	0 (0.0); [0–10.3]
Uncommon	0 (0.0); [0–10.3]
Rare	0 (0.0); [0–10.3]
Never observed	36 (100.0); [70.0–100]
Q15. Cramps at the injection site	
Very frequent	0 (0.0); [0–10.3]
Frequent	0 (0.0); [0–10.3]
Uncommon	1 (2.8); [0–10.3]
Rare	3 (8.3); [1.8–24.4]
Never observed	32 (88.9); [60.8–100]
Q16. Sensitivity at the treated area	
Very frequent	0 (0.0); [0–10.3]
Frequent	2 (5.6); [0.7–20.1]
Uncommon	5 (13.9); [4.5–32.4]
Rare	9 (25.0); [11.4–47.5]
Never observed	20 (55.6); [33.9–85.8]
Q17. Ischemic event	
Very frequent	0 (0.0); [0–10.3]
Frequent	0 (0.0); [0–10.3]
Uncommon	0 (0.0); [0–10.3]
Rare	0 (0.0); [0–10.3]
Never observed	36 (100.0); [70.0–100]
Q18. Visual impairment/blindness^a^	
Very frequent	0 (0.0); [0–10.5]
Frequent	0 (0.0); [0–10.5]
Uncommon	0 (0.0); [0–10.5]
Rare	0 (0.0); [0–10.5]
Never observed	35 (100.0); [70.0–100]

*Note:* 95% CI is expressed as %.

Abbreviations: 95% CI, 95% confidence interval; PLLA, poly‐L‐lactic acid.

^a^ One of the participants did not respond to that question due to technical issues.

The AEs reported in the survey have been previously described in various studies [2, 3, 4, 5, 6]. In fact, the survey results align with the existing literature, indicating that most patients did not experience any AEs.

One of the most common concerns with PLLA fillers is the potential formation of nodules or lumps under the skin, particularly if the filler is injected improperly or in excessive amounts. However, as shown in the published evidence [2, 3, 4, 5, 6] and in this survey, the incidence of nodules (either early or late onset has been perceived as extremely rare or absent).

We acknowledge that this study has several limitations. These include potential sampling bias, as the selected clinics and professionals may not fully represent the broader population. Nevertheless, they represent all the specialists who currently have experience with the new PLLA‐LaSynPro filler. Additionally, the limited duration of experience with this new PLLA‐LaSynPro filler may influence perceptions, which could change with a larger patient sample or longer follow‐up period.

In summary, according to the survey results, 20%–30% of participants reported that mild AEs occur frequently or very frequently. Most respondents noted that moderate AEs were infrequent or rare. Importantly, nearly all respondents reported never having observed severe AEs. These findings, derived from self‐reported perceptions of experienced practitioners, aligned with a favorable safety profile for the new PLLA‐LaSynPro filler. Nevertheless, given the subjective nature of the assessment and the absence of objective clinical outcome data, conclusions regarding the frequency of moderate to severe AEs should be made with caution. The safety‐related descriptors presented reflect qualitative impressions rather than quantified incidence rates and should not be construed as definitive clinical evidence.

## Author Contributions

M.S.‐C. designed and directed the project, the main conceptual ideas, and proof outline. P.C.R. and F.R.T. drafted the manuscript, literature search, and designed the tables and figures. M.S.‐C., P.C.R., F.R.T., C.C.T., A.R.S., and V.P.R. collected data. F.R.T. and A.R.S. provided visualization and data curation. V.P.R. contributed to methodology, main conceptual ideas, and critical review and edition of the manuscript. All authors reviewed the results and approved the final version of the manuscript.

## Conflicts of Interest

Dr. Maribel Serrano‐Coronado and Dr. Victoria Páez Ruiz received research grants to cover the costs of **medical** writing services and publication fees, honoraria for lectures, and travel support to attend educational meetings from Nordberg Medical. Paula Catena Rallo, Francisca Rubio Toral, Cristina Chantada Tirado and Adriana Ribé Subirà has no financial interests to declare.

## Supporting information


**Data S1:** jocd70439‐sup‐0001‐DataS1.docx.

## Data Availability

The data that support the findings of this study are available on request from the corresponding author. The data are not publicly available due to privacy or ethical restrictions.
