# Supplementary material for: Clinical Perspectives on the Safety Profile of Poly‐L‐Lactic Acid (Juläine): Results From a National Survey
Source: J Cosmet Dermatol. 2025 Sep 8;24(9):e70439. doi: 10.1111/jocd.70439 (PMC12415935; doi:10.1111/jocd.70439)
Supplement: Supplementary file 1 — Data S1: jocd70439‐sup‐0001‐DataS1.docx. [file JOCD-24-e70439-s001.docx]

**Clinical Perspectives on the Safety Profile of Poly-L-Lactic Acid (Juläine™): Results from a National Survey**

**Supplementary material**

Study Methodology

*Study Design and Sample*

This study, conducted between September and November 2024, employed a descriptive, cross-sectional survey design. The target population consisted of aesthetic medicine practitioners with experience using the new poly-L-lactic acid (PLLA)-LASYNPRO^TM^ (Juläine^TM^, Nordberg Medical AB, Sweden).

*Ethical considerations*

The Ethical Review Board of the " Clínica Tufet"(Barcelona, Spain) advised that no formal ethical approval was required. Nevertheless, all participants gave their consent to participate in the survey and agreed to the publication of the survey results. All questionnaire responses were kept confidential, and no associations were made between the results and any specific practitioner.

*Study road-map*

A multidisciplinary team of specialists in aesthetic medicine conceptualized and developed the study. During an initial virtual meeting in September 5, 2024, the panel agreed to conduct a comprehensive, non-systematic review of the available scientific evidence regarding the safety profile of the new PLLA-LASYNPRO^TM^ filler in aesthetic medicine.

After identifying the main adverse events, an 18-question survey was created to assess prescribing physicians’ perceptions of their clinical experience and the likelihood of these adverse events occurring. The survey was designed in Spanish, as the study was initially planned to be conducted only in Spain (Table S1 presents the English version of the survey).

During the second phase of the project, which took place between October 3 and 11, potential survey participants were contacted and sent the questionnaire.

The survey was distributed to 48 centers/specialists. The participants were notified about the voluntary nature of participation, confidentiality, and non-compensation for participation.

The deadline for questionnaire responses was set for October 22. Following this, a second virtual meeting took place on October 29, during which the panel members discussed the survey results.

**PLLA-LASYNPRO™ Product Characteristics and Reconstitution**

PLLA-LASYNPRO™ represents a next-generation poly-L-lactic acid (PLLA) injectable biostimulant, specifically engineered to promote extracellular matrix (ECM) regeneration and stimulate neocollagenesis. In this context, PLLA-LASYNPRO™ is intended to potentially engage low-inflammatory, bioinductive pathways, rather than predominantly provoking a foreign-body reaction, as has been reported with certain first-generation PLLA products (1-4). This bioengineering approach aims to promote tissue remodeling while preserving native tissue architecture and minimizing immune system activation (1-4).

Morphologically, PLLA-LASYNPRO™ consists of smooth, spherical microspheres with a highly uniform size distribution and low porosity (5). This contrasts sharply with the irregular, porous morphology observed in earlier PLLA formulations (5), with a size of 32.4µm (Data on file), composed of 150mg of poly-L-lactic acid microspheres, 45mg of sodium carboxymethylcellulose and 145mg of non-pyrogenic mannitol.

For preparation, PLLA-LASYNPRO™ is reconstituted with 5 mL of sterile, physiologically balanced saline solution for injection, following the manufacturer's Instructions for Use (6). The saline is introduced into the vial and the suspension is agitated vigorously for approximately one minute to ensure complete and homogeneous reconstitution (5,6).

*Data analysis*

Data were analyzed by using MedCalc® Statistical Software version 23.1.5 (MedCalc Software Ltd, Ostend, Belgium; https://www.medcalc.org; 2025). Before the study, it was determined that at least 37 practitioners need to answer the questionnaire, at a significance level of 0.05, and a margin of error of 10%.

Data analysis was performed by an independent statistician, who was blind for the question and response options.

**References**

1. Zhu W, Dong C. Poly-L-Lactic acid increases collagen gene expression and synthesis in cultured dermal fibroblast (Hs68) through the TGF-β/Smad pathway. J Cosmet Dermatol. 2023;22(4):1213-1219.

2. Kim SA, Kim HS, Jung JW, Suh SI, Ryoo YW. Poly-L-Lactic Acid Increases Collagen Gene Expression and Synthesis in Cultured Dermal Fibroblast (Hs68) Through the p38 MAPK Pathway. Ann Dermatol. 2019;31(1):97-100.

3. Bertossi D, Cavallini M, Camporese A, Dell'Avanzato R, Kefalas N, Massidda E, et al. First Insights on the Upcoming Role of Next-Generation PLLA-LASYNPRO™ in Aesthetic and Regenerative Medicine. A Survey of Experts — Clinical Practice Suggestions. *Preprints* 2025, 2025040295. <https://doi.org/10.20944/preprints202504.0295.v1>. Available in: <https://www.preprints.org/frontend/manuscript/485159b1e538f3c7cd234bf5d0cb0c27/download_pub> Last accessed July 1, 2025.

4. Zou Y, Cao M, Tai M, Zhou H, Tao L, Wu S, et al. A Feedback Loop Driven by H4K12 Lactylation and HDAC3 in Macrophages Regulates Lactate-Induced Collagen Synthesis in Fibroblasts Via the TGF-β Signaling. Adv Sci (Weinh). 2025;12(13):e2411408. doi: 10.1002/advs.202411408.

5. Kubik P, Gruszczyński W, Filipowska M. Comparative Analysis of Reconstitution and Solubility of Two Poly-L-Lactic Acid Fillers for Medical Applications. *Polymers*. 2025; 17(13):1778. <https://doi.org/10.3390/polym17131778>.

6. Juläine. Instructions for Use. Available online: https://julaineofsweden.com/wp-content/uploads/2024/06/Instructions-for-Use-JULAINE-of-Sweden.pdf Last accessed July 1, 2025.

Table S1. Questionnaire

| **Question** | **Possible answers** |
| --- | --- |
| **Q1. Time of experience with PLLA, months** | < 3  3 – 6  > 6 |
| **Q2. Hyperpigmentation** | Very frequent  Frequent  Uncommon  Rare  Never seen |
| **Q3. Loss of pigmentation** | Very frequent  Frequent  Uncommon  Rare  Never seen |
| **Q4. Short-duration edema (<1 week)** | Very frequent  Frequent  Uncommon  Rare  Never seen |
| **Q5. Long-duration edema (>1 week)** | Very frequent  Frequent  Uncommon  Rare  Never seen |
| **Q6. Short-duration pain (<1 week)** | Very frequent  Frequent  Uncommon  Rare  Never seen |
| **Q7. Infection** | Very frequent  Frequent  Uncommon  Rare  Never seen |
| **Q8. Mild bleeding** | Very frequent  Frequent  Uncommon  Rare  Never seen |
| **Q9. Redness** **/ Erythema** | Very frequent  Frequent  Uncommon  Rare  Never seen |
| **Q10. Bruising/Hematomas** | Very frequent  Frequent  Uncommon  Rare  Never seen |
| **Q11. Ulceration** | Very frequent  Frequent  Uncommon  Rare  Never seen |
| **Q12. Itching** | Very frequent  Frequent  Uncommon  Rare  Never seen |
| **Q13. Early onset nodules (0-3 months)** | Very frequent  Frequent  Uncommon  Rare  Never seen |
| **Q14. Late onset nodules (>3 months)** | Very frequent  Frequent  Uncommon  Rare  Never seen |
| **Q15. Cramps at the injection site** | Very frequent  Frequent  Uncommon  Rare  Never seen |
| **Q16. Sensitivity at the treated area** | Very frequent  Frequent  Uncommon  Rare  Never seen |
| **Q17. Ischemic event** | Very frequent  Frequent  Uncommon  Rare  Never seen |
| **Q18. Visual impairment/blindness** | Very frequent  Frequent  Uncommon  Rare  Never seen |

PLLA: Poly-L-lactic acid.

Clarification: The descriptors “Very frequent”; “frequent”; Uncommon”; “rare”; and “Never seen” used in this section reflect the subjective terminology provided by survey respondents and should be interpreted solely as qualitative impressions rather than quantitative measures of incidence; they do not constitute objective clinical evidence.
